# Perception Sensors for Road Applications

**DOI:** 10.3390/s19235294

**Published:** 2019-12-01

**Authors:** Felipe Jiménez

**Affiliations:** Instituto Universitario de Investigación del Automóvil (INSIA), Universidad Politécnica de Madrid, 28031 Madrid, Spain; felipe.jimenez@upm.es

## 1. Introduction

New assistance systems and the applications of autonomous driving of road vehicles imply ever-greater requirements for perception systems that are necessary in order to increase the robustness of decisions and to avoid false positives or false negatives.

In this sense, there are many technologies that can be used, both in vehicles and infrastructure. In the first case, technology, such as LiDAR or computer vision, is the basis for growth in the automation levels of vehicles, although its actual deployment also demonstrates the problems that can be found in real scenarios and that must be solved to continue on the path of improving the safety and efficiency of road traffic.

Usually, given the limitations of each of the technologies, it is common to resort to sensorial fusion, both of the same of type sensors and of different types.

Additionally, obtaining data for decision-making does not only come from on-board sensors, but wireless communications with the outside world allow vehicles to offer greater electronic horizons. In the same way, positioning in precise and detailed digital maps provides additional information that can be very useful in interpreting the environment.

The sensors also cover the driver in order to analyze their ability to perform tasks safely.

In all areas, it is crucial to study the limitations of each of the solutions and sensors, as well as to establish tools that try to alleviate these issues, either through improvements in hardware or in software. In this sense, the specifications requested of sensors must be established and specific methods must be developed to validate said specifications for the sensors and to complete the systems.

In conclusion, this Special Issue aims to bring together innovative developments in areas related to sensors in vehicles and the use of the information for assistance systems and autonomous vehicles.

## 2. Papers in the Special Issue

As assistance and automation increase in road vehicles, the requirements of perception systems rise significantly and new solutions emerge in research and the market. Reference [1] presents a systematic review of the perception systems and simulators for autonomous vehicles. This work has been divided into three parts. In the first part, perception systems are categorized as environment perception systems and positioning estimation systems. In the second part, the main elements to be taken into account in the simulation of a perception system of an AV are presented. Finally, the current state of regulations that are being applied in different countries around the world, on issues concerning the implementation of autonomous vehicles, is presented.

As previously mentioned, the number of small sophisticated wireless sensors that share the electromagnetic spectrum is expected to grow rapidly over the next decade and interference between these sensors is anticipated to become a major challenge. In Reference [2], the interference mechanisms in one such sensor, automotive radars, is studied, and the results are directly applicable to a range of other sensor situations.

One of the most common applications of perception systems in vehicles is obstacle detection. In this field, several technologies have been used for years and new algorithms have tried to obtain more robust and efficient results under complex scenarios.

A robust Multiple Object Detection and Tracking (MODT) algorithm for a non-stationary base is presented in Reference [3], using multiple 3D LiDARs for perception. The merged LiDAR data is treated with an efficient MODT framework, considering the limitations of the vehicle-embedded computing environment. The ground classification is obtained through a grid-based method, while considering a non-planar ground. Furthermore, unlike prior works, a 3D grid-based clustering technique is developed to detect objects under elevated structures. The centroid measurements obtained from the object detection are tracked using an Interactive Multiple Model-Unscented Kalman Filter–Joint Probabilistic Data Association Filter.

Reference [4] presents an efficient moving object detection algorithm that can cope with moving camera environments. In addition, a hardware design and the implementation results for the real-time processing of the proposed algorithm are presented. The proposed moving object detector was designed using hardware description language (HDL) and its real-time performance was evaluated using an FPGA based test system.

A computationally low-cost and robust detecting and tracking moving objects (DATMO) system, which uses as input only 2D laser rangefinder information, is presented in Reference [5]. Due to its low requirements, both in sensor needs and computation, the DATMO algorithm is meant to be used in current autonomous guided vehicles to improve their reliability for the cargo transportation tasks at port terminals, advancing towards the next generation of fully autonomous transportation vehicles.

A continuous waveform radar is widely used in intelligent transportation systems. There are several waveforms and the chirp sequence waveform has the ability to extract the range and velocity parameters of multiple targets. Reference [6] proposes a new waveform that follows the practical application requirements, high precision requirements, and low system complexity requirements. Theoretical analysis and simulation results verify that the new radar waveform is capable of measuring the range and radial velocity simultaneously and unambiguously, with high accuracy and resolution even in multi-target situations.

Another classical use of perception systems is the characterization of the scenario and the road. An Extended Line Map (ELM)-based precise vehicle localization method is proposed in Extended Line Map [7], and is implemented using 3D Light Detection and Ranging (LIDAR). A binary occupancy grid map in which grids for road marking or vertical structures have a value of one and the rest have a value of zero was created using the reflectivity and distance data of the 3D LIDAR. 

Furthermore, vision-based lane-detection methods provide low-cost density information about roads. A robust and efficient method to expand the application of these methods to cover low-speed environments is presented in Reference [8].

Moreover, perception sensors are also used for driver and other passenger detection and characterization. Perhaps the least intrusive, physiology-based approach is to remotely monitor driver drowsiness by using cameras to detect facial expressions. A multi-timescale drowsiness characterization system composed of four binary drowsiness classifiers, operating at four distinct timescales and trained jointly, was developed in Reference [9].

Finally, the information retrieved by perception sensors can be used for decision-making systems. The first step for decision-making in an autonomous vehicle or an assistance system is the understanding of the environment. Reference [10] presents three ways of modelling traffic in a roundabout, quite a critical scenario, based on: (i) The roundabout geometry; (ii) mean path taken by vehicles inside the roundabout; and (iii) a set of reference trajectories traversed by vehicles inside the roundabout.

Reference [11] presents a machine learning-based technique to build a predictive model and to generate rules of action to allow autonomous vehicles to perform roundabout maneuvers. The approach consists of building a predictive model of vehicle speeds and steering angles based on collected data that are related to driver–vehicle interactions and other aggregated data intrinsic to the traffic environment.

Reference [12] presents a path-planning algorithm based on potential fields. Potential models are adjusted so that their behavior is appropriate to the environment and the dynamics of the vehicle and they can face almost any unexpected scenarios. The response of the system considers the road characteristics (e.g., maximum speed, lane line curvature, etc.) and the presence of obstacles and other users.
